# Supplementary material for: A hierarchical model of daily stream temperature using air-water temperature synchronization, autocorrelation, and time lags
Source: PeerJ. 2016 Feb 29;4:e1727. doi: 10.7717/peerj.1727 (PMC4782734; doi:10.7717/peerj.1727)
Supplement: Supplemental Information 1 [file peerj-04-1727-s001.docx]

**Supplemental Information**

Table S1. Parameter r-hat values, means, and credible intervals

| Parameter | Rhat | Mean | sd | 2.5% CI | 25% CI | 75% CI | 97.5% CI |
| --- | --- | --- | --- | --- | --- | --- | --- |
| B[0] | 1.000 | 15.090 | 0.177 | 14.741 | 14.978 | 15.201 | 15.449 |
| B[1] | 1.000 | 1.520 | 0.027 | 1.468 | 1.501 | 1.539 | 1.573 |
| B[2] | 1.000 | 0.199 | 0.016 | 0.170 | 0.188 | 0.210 | 0.231 |
| B[3] | 1.001 | 0.154 | 0.016 | 0.122 | 0.144 | 0.165 | 0.185 |
| B[4] | 1.000 | 0.360 | 0.015 | 0.331 | 0.349 | 0.371 | 0.391 |
| B[5] | 1.000 | -0.129 | 0.012 | -0.152 | -0.137 | -0.121 | -0.106 |
| B[6] | 1.000 | -0.503 | 0.082 | -0.667 | -0.559 | -0.447 | -0.344 |
| B[7] | 1.000 | 0.592 | 0.088 | 0.418 | 0.533 | 0.651 | 0.760 |
| B[8] | 1.000 | -0.545 | 0.079 | -0.699 | -0.598 | -0.491 | -0.393 |
| B[9] | 1.000 | 0.217 | 0.039 | 0.140 | 0.190 | 0.243 | 0.295 |
| B[10] | 1.000 | 0.187 | 0.040 | 0.110 | 0.159 | 0.214 | 0.265 |
| B[11] | 1.000 | 0.101 | 0.038 | 0.026 | 0.076 | 0.127 | 0.177 |
| B.year[1,1] | 1.001 | 0.321 | 0.276 | -0.228 | 0.140 | 0.502 | 0.873 |
| B.year[2,1] | 1.000 | -0.652 | 0.279 | -1.205 | -0.835 | -0.469 | -0.104 |
| B.year[3,1] | 1.000 | -0.415 | 0.276 | -0.991 | -0.593 | -0.227 | 0.104 |
| B.year[4,1] | 1.000 | -0.122 | 0.245 | -0.606 | -0.292 | 0.045 | 0.338 |
| B.year[5,1] | 1.001 | -0.413 | 0.201 | -0.806 | -0.543 | -0.279 | -0.009 |
| B.year[6,1] | 1.001 | -0.195 | 0.199 | -0.587 | -0.328 | -0.062 | 0.199 |
| B.year[7,1] | 1.002 | -0.023 | 0.209 | -0.445 | -0.159 | 0.112 | 0.399 |
| B.year[8,1] | 1.002 | -0.304 | 0.229 | -0.760 | -0.452 | -0.152 | 0.136 |
| B.year[9,1] | 1.000 | -0.145 | 0.227 | -0.588 | -0.297 | 0.003 | 0.291 |
| B.year[10,1] | 1.000 | -0.209 | 0.213 | -0.637 | -0.351 | -0.073 | 0.204 |
| B.year[11,1] | 1.000 | -0.564 | 0.217 | -1.001 | -0.706 | -0.418 | -0.151 |
| B.year[12,1] | 1.000 | 1.036 | 0.199 | 0.656 | 0.907 | 1.166 | 1.436 |
| B.year[13,1] | 1.000 | 0.492 | 0.209 | 0.078 | 0.355 | 0.623 | 0.914 |
| B.year[14,1] | 1.000 | 0.592 | 0.204 | 0.193 | 0.451 | 0.731 | 0.991 |
| B.year[15,1] | 1.000 | 0.621 | 0.214 | 0.222 | 0.476 | 0.764 | 1.042 |
| B.year[1,2] | 1.000 | 1.308 | 0.313 | 0.695 | 1.102 | 1.520 | 1.921 |
| B.year[2,2] | 1.000 | 1.084 | 0.295 | 0.503 | 0.891 | 1.279 | 1.659 |
| B.year[3,2] | 1.000 | 1.145 | 0.294 | 0.552 | 0.952 | 1.344 | 1.713 |
| B.year[4,2] | 1.000 | 2.150 | 0.294 | 1.586 | 1.951 | 2.345 | 2.724 |
| B.year[5,2] | 1.000 | 2.181 | 0.213 | 1.762 | 2.033 | 2.324 | 2.603 |
| B.year[6,2] | 1.000 | 1.223 | 0.180 | 0.869 | 1.102 | 1.347 | 1.567 |
| B.year[7,2] | 1.000 | 1.784 | 0.210 | 1.370 | 1.645 | 1.922 | 2.188 |
| B.year[8,2] | 1.000 | 1.341 | 0.249 | 0.852 | 1.176 | 1.514 | 1.829 |
| B.year[9,2] | 1.000 | 1.629 | 0.246 | 1.143 | 1.471 | 1.792 | 2.101 |
| B.year[10,2] | 1.000 | 1.395 | 0.219 | 0.967 | 1.245 | 1.538 | 1.834 |
| B.year[11,2] | 1.001 | 0.850 | 0.198 | 0.465 | 0.715 | 0.986 | 1.236 |
| B.year[12,2] | 1.000 | 1.682 | 0.161 | 1.364 | 1.570 | 1.794 | 1.986 |
| B.year[13,2] | 1.000 | 1.491 | 0.181 | 1.142 | 1.371 | 1.614 | 1.842 |
| B.year[14,2] | 1.000 | 1.307 | 0.192 | 0.929 | 1.178 | 1.441 | 1.682 |
| B.year[15,2] | 1.000 | 1.471 | 0.217 | 1.038 | 1.327 | 1.614 | 1.895 |
| B.year[1,3] | 1.000 | -2.504 | 0.149 | -2.795 | -2.604 | -2.403 | -2.218 |
| B.year[2,3] | 1.000 | -2.056 | 0.129 | -2.307 | -2.145 | -1.971 | -1.794 |
| B.year[3,3] | 1.000 | -2.527 | 0.191 | -2.909 | -2.651 | -2.400 | -2.151 |
| B.year[4,3] | 1.000 | -2.296 | 0.137 | -2.569 | -2.386 | -2.205 | -2.026 |
| B.year[5,3] | 1.000 | -2.517 | 0.109 | -2.728 | -2.591 | -2.445 | -2.295 |
| B.year[6,3] | 1.001 | -2.370 | 0.087 | -2.543 | -2.428 | -2.312 | -2.195 |
| B.year[7,3] | 1.000 | -2.252 | 0.105 | -2.455 | -2.325 | -2.181 | -2.051 |
| B.year[8,3] | 1.002 | -2.226 | 0.132 | -2.481 | -2.313 | -2.137 | -1.964 |
| B.year[9,3] | 1.000 | -2.272 | 0.134 | -2.536 | -2.362 | -2.179 | -2.013 |
| B.year[10,3] | 1.000 | -2.522 | 0.130 | -2.767 | -2.614 | -2.434 | -2.268 |
| B.year[11,3] | 1.000 | -1.981 | 0.093 | -2.167 | -2.044 | -1.918 | -1.798 |
| B.year[12,3] | 1.000 | -2.422 | 0.073 | -2.567 | -2.471 | -2.369 | -2.279 |
| B.year[13,3] | 1.000 | -2.387 | 0.092 | -2.565 | -2.449 | -2.325 | -2.208 |
| B.year[14,3] | 1.000 | -1.918 | 0.091 | -2.098 | -1.981 | -1.859 | -1.743 |
| B.year[15,3] | 1.000 | -2.447 | 0.107 | -2.657 | -2.520 | -2.373 | -2.241 |
| B.year[1,4] | 1.000 | -0.202 | 0.138 | -0.471 | -0.292 | -0.108 | 0.067 |
| B.year[2,4] | 1.000 | -0.153 | 0.109 | -0.369 | -0.227 | -0.079 | 0.062 |
| B.year[3,4] | 1.000 | 0.195 | 0.138 | -0.063 | 0.101 | 0.284 | 0.473 |
| B.year[4,4] | 1.000 | -0.486 | 0.130 | -0.739 | -0.573 | -0.398 | -0.239 |
| B.year[5,4] | 1.000 | -0.430 | 0.135 | -0.694 | -0.522 | -0.337 | -0.172 |
| B.year[6,4] | 1.000 | 0.069 | 0.089 | -0.101 | 0.008 | 0.131 | 0.240 |
| B.year[7,4] | 1.000 | -0.096 | 0.104 | -0.293 | -0.165 | -0.027 | 0.111 |
| B.year[8,4] | 1.000 | -0.251 | 0.134 | -0.505 | -0.343 | -0.159 | 0.017 |
| B.year[9,4] | 1.000 | 0.199 | 0.146 | -0.086 | 0.102 | 0.294 | 0.492 |
| B.year[10,4] | 1.000 | 0.040 | 0.132 | -0.217 | -0.048 | 0.132 | 0.298 |
| B.year[11,4] | 1.001 | 0.103 | 0.077 | -0.049 | 0.050 | 0.155 | 0.253 |
| B.year[12,4] | 1.000 | -0.382 | 0.057 | -0.491 | -0.421 | -0.343 | -0.272 |
| B.year[13,4] | 1.000 | -0.086 | 0.079 | -0.238 | -0.141 | -0.031 | 0.064 |
| B.year[14,4] | 1.000 | -0.248 | 0.083 | -0.410 | -0.303 | -0.191 | -0.086 |
| B.year[15,4] | 1.000 | -0.101 | 0.105 | -0.302 | -0.171 | -0.031 | 0.111 |
| ar1[1] | 1.000 | 0.789 | 0.012 | 0.765 | 0.781 | 0.797 | 0.814 |
| ar1[2] | 1.000 | 0.771 | 0.018 | 0.734 | 0.759 | 0.784 | 0.800 |
| ar1[3] | 1.001 | 0.801 | 0.013 | 0.777 | 0.791 | 0.810 | 0.827 |
| ar1[4] | 1.000 | 0.767 | 0.019 | 0.726 | 0.755 | 0.781 | 0.799 |
| ar1Mean | 1.001 | 0.788 | 0.038 | 0.734 | 0.773 | 0.795 | 0.907 |
| ar1SD | 1.005 | 0.047 | 0.063 | 0.002 | 0.014 | 0.050 | 0.245 |
| mu.year[1] | NA | 0.000 | 0.000 | 0.000 | 0.000 | 0.000 | 0.000 |
| mu.year[2] | 1.001 | 1.471 | 0.153 | 1.173 | 1.370 | 1.569 | 1.786 |
| mu.year[3] | 1.001 | -2.314 | 0.103 | -2.517 | -2.382 | -2.246 | -2.118 |
| mu.year[4] | 1.000 | -0.123 | 0.100 | -0.318 | -0.189 | -0.060 | 0.076 |
| sigma | 1.000 | 0.597 | 0.004 | 0.588 | 0.594 | 0.600 | 0.606 |
| sigma.b.year[1] | 1.000 | 0.580 | 0.123 | 0.394 | 0.494 | 0.646 | 0.861 |
| sigma.b.year[2] | 1.000 | 0.501 | 0.113 | 0.327 | 0.420 | 0.563 | 0.764 |
| sigma.b.year[3] | 1.000 | 0.349 | 0.070 | 0.239 | 0.300 | 0.388 | 0.516 |
| sigma.b.year[4] | 1.000 | 0.357 | 0.071 | 0.245 | 0.307 | 0.397 | 0.527 |

Figure S1. Violin plots of monthly daily mean water temperatures across years (panels) and sites. Filled circles show mean for each month, open circles show means for each month for each stream, and lines show the mean for the month, stream, year combination.





Figure S2. Observed vs. predicted daily water temperatures for combinations of site and year. The line in each panel is the 1:1 line.


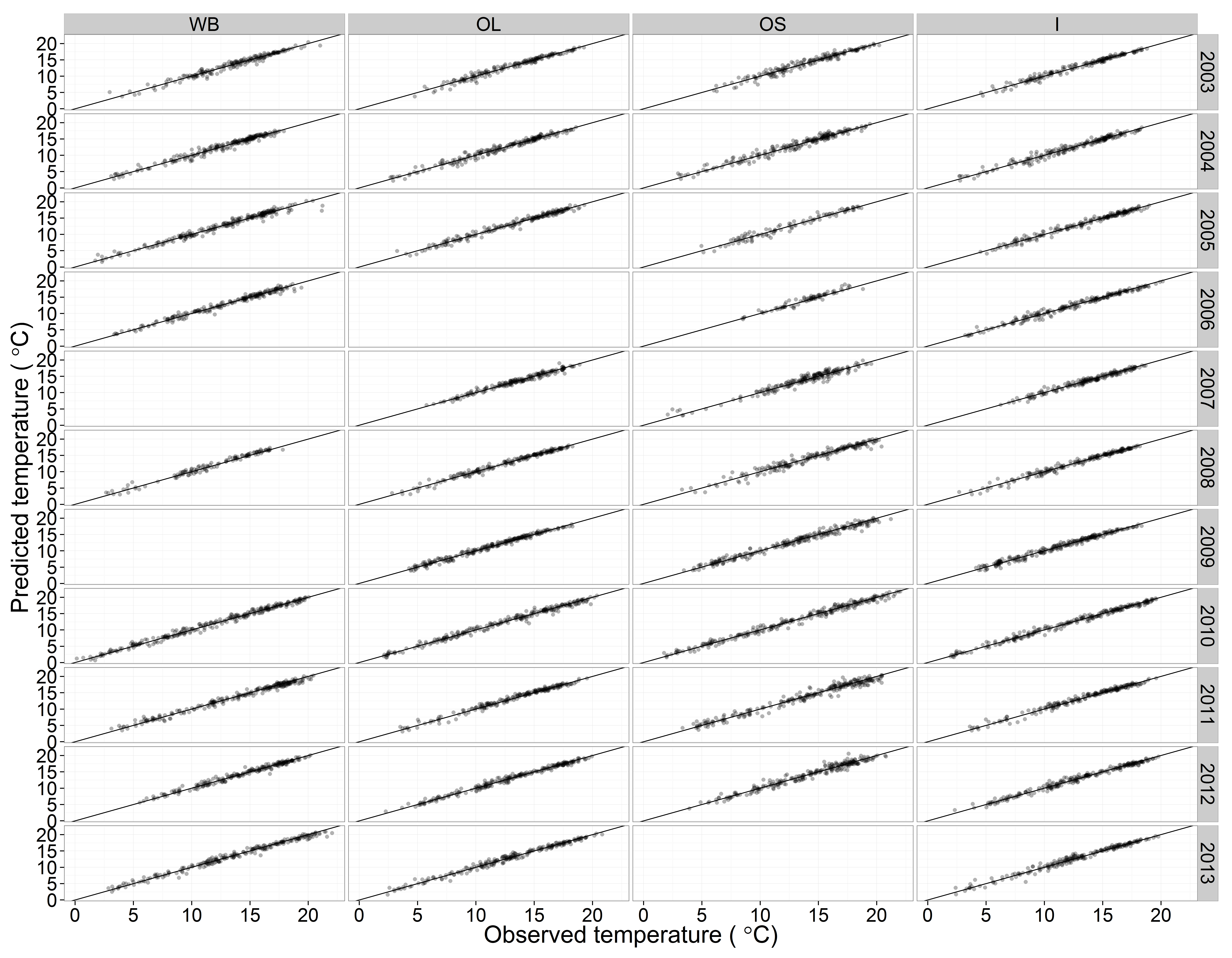


Figure S3. Root mean square error of the test data set (triangles) and training data set (circles) for 10 replicates of runs with increasing proportions of data left out of the training set.


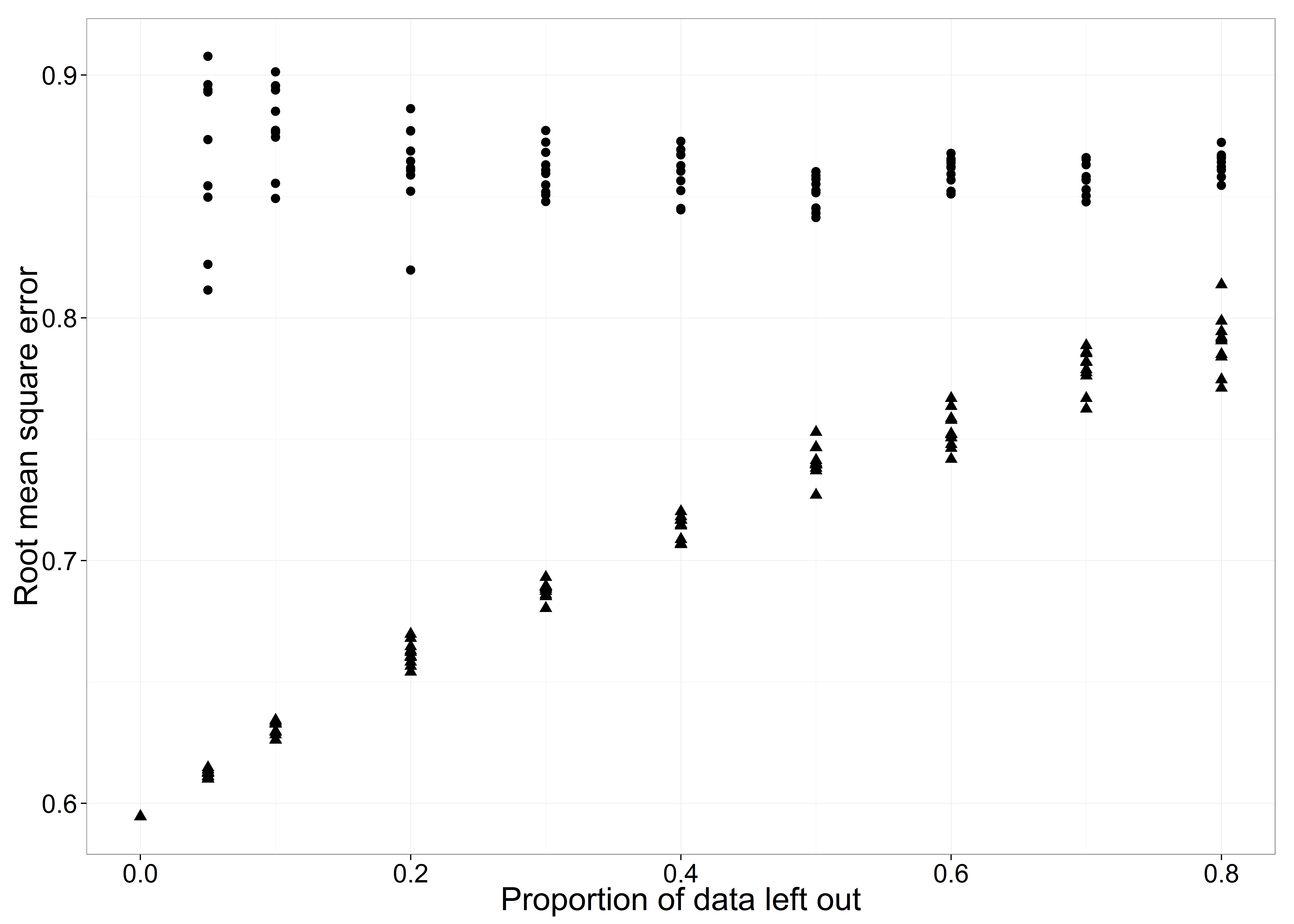


Figure S4. Predicted cubic curves for each year against day of year (Eq. 7). The white dots signify the yearly maximum water temperature and the day of maximum water temperature.


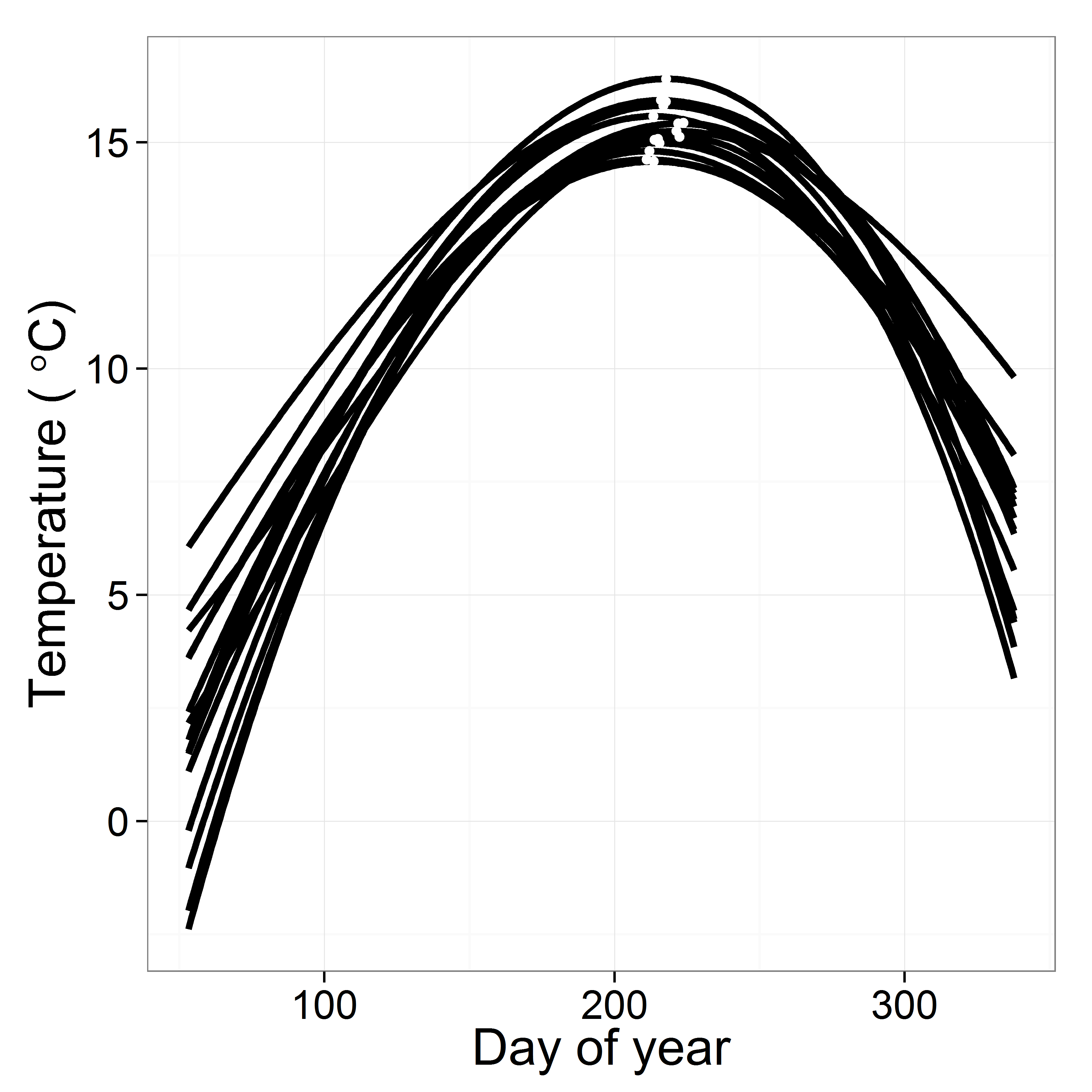


JAGS model code for the stream temperature model.

model{

# Temperature input data are in variable ‘temp’

# Temperature data are indexed by ‘firstObsRows’ and ‘evalRows’. ‘firstObsRows’ indicates rows in the input data frame that are the first observation in a series (no ar1 term) and ‘evalRows’ indicates rows that are evaluated with the ar1 term. Temperature data are also indexed by ‘river’ and ‘year’

# ‘trend’ indicates the fixed effects and ar1 indicates the ar1 effects

####################################

# Days without an observation on the previous day (first observation in a series)

# No autoregressive term

for( i in 1:nFirstObsRows ){

temp[ firstObsRows[i] ] ~ dnorm( stream.mu[ firstObsRows[i] ], tau )

stream.mu[ firstObsRows[i] ] <- trend[ firstObsRows[i] ]

trend[ firstObsRows[i] ] <- inprod( B.0[], X.0[ firstObsRows[i], ] ) + inprod( B.year[year[ firstObsRows[i]], ], X.year[ firstObsRows[i], ] )

}

####################################

# Days with an observation on the previous day (not the first observation in a series)

# With autoregressive term (ar1)

for( i in 1:nEvalRows ){ # n observations

temp[ evalRows[i] ] ~ dnorm( stream.mu[ evalRows[i] ], tau )

# the mean is a combination of the trend and the autoregressive parts

stream.mu[ evalRows[i] ] <- trend[ evalRows[i] ] + ar1[ river[ evalRows[i] ] ] * ( temp[ evalRows[i]-1 ] - trend[ evalRows[i]-1 ] )

trend[ evalRows[i] ] <- inprod( B.0[], X.0[ evalRows[i], ] ) + inprod( B.year[ year[ evalRows[i]], ], X.year[ evalRows[i], ] )

}

####################################

# prior for ar1

for( i in 1:nRiver ){

ar1[i] ~ dnorm( ar1Mean, pow( ar1SD,-2 ) ) T( -1,1 )

}

ar1Mean ~ dunif( -1, 1 )

ar1SD ~ dunif( 0, 2 )

####################################

# prior for model variance

sigma ~ dunif( 0, 100 )

tau <- pow( sigma, -2 )

####################################

# priors on coefs for fixed effect predictors

for( k in 1:K.0 ){

B.0[k] ~ dnorm( 0, 0.001 )

}

####################################

# YEAR EFFECTS

# Priors for random effects of year

for( t in 1:Ti ){ # Ti years

B.year[ t, 1:L ] ~ dmnorm( mu.year[ ], tau.B.year[ , ] )

}

mu.year[1] <- 0

for( l in 2:L ){

mu.year[l] ~ dnorm( 0, 0.0001 )

}

# Prior on multivariate normal std deviation

tau.B.year[ 1:L, 1:L ] ~ dwish( W.year[ , ], df.year )

df.year <- L + 1

sigma.B.year[ 1:L, 1:L ] <- inverse( tau.B.year[ , ] )

for( l in 1:L ){

for( l.prime in 1:L ){

rho.B.year[ l, l.prime ] <- sigma.B.year[ l, l.prime]/sqrt(sigma.B.year[l, l ]*sigma.B.year[ l.prime, l.prime ])

}

sigma.b.year[l] <- sqrt( sigma.B.year[l, l] )

}
