# Supplementary material for: A hierarchical model of daily stream temperature using air-water temperature synchronization, autocorrelation, and time lags
Source: PeerJ. 2016 Feb 29;4:e1727. doi: 10.7717/peerj.1727 (PMC4782734; doi:10.7717/peerj.1727)
Supplement: Table S1 [file peerj-04-1727-s003.docx]

Table S1. Parameter r-hat values, means, and credible intervals.

| Parameter | Rhat | Mean | sd | 2.5% CI | 25% CI | 75% CI | 97.5% CI |
| --- | --- | --- | --- | --- | --- | --- | --- |
| B[0], Intercept | 1.000 | 15.090 | 0.177 | 14.741 | 14.978 | 15.201 | 15.449 |
| B[1], Temperature (T) | 1.000 | 1.520 | 0.027 | 1.468 | 1.501 | 1.539 | 1.573 |
| B[2], T(d-1) | 1.000 | 0.199 | 0.016 | 0.170 | 0.188 | 0.210 | 0.231 |
| B[3], T(d-2) | 1.001 | 0.154 | 0.016 | 0.122 | 0.144 | 0.165 | 0.185 |
| B[4], Flow (F) | 1.000 | 0.360 | 0.015 | 0.331 | 0.349 | 0.371 | 0.391 |
| B[5], T * F | 1.000 | -0.129 | 0.012 | -0.152 | -0.137 | -0.121 | -0.106 |
| B[6], OL | 1.000 | -0.503 | 0.082 | -0.667 | -0.559 | -0.447 | -0.344 |
| B[7], OS | 1.000 | 0.592 | 0.088 | 0.418 | 0.533 | 0.651 | 0.760 |
| B[8], IL | 1.000 | -0.545 | 0.079 | -0.699 | -0.598 | -0.491 | -0.393 |
| B[9], OL*T | 1.000 | 0.217 | 0.039 | 0.140 | 0.190 | 0.243 | 0.295 |
| B[10], OS * T | 1.000 | 0.187 | 0.040 | 0.110 | 0.159 | 0.214 | 0.265 |
| B[11], IL * T | 1.000 | 0.101 | 0.038 | 0.026 | 0.076 | 0.127 | 0.177 |
| B.year[1,1] | 1.001 | 0.321 | 0.276 | -0.228 | 0.140 | 0.502 | 0.873 |
| B.year[2,1] | 1.000 | -0.652 | 0.279 | -1.205 | -0.835 | -0.469 | -0.104 |
| B.year[3,1] | 1.000 | -0.415 | 0.276 | -0.991 | -0.593 | -0.227 | 0.104 |
| B.year[4,1] | 1.000 | -0.122 | 0.245 | -0.606 | -0.292 | 0.045 | 0.338 |
| B.year[5,1] | 1.001 | -0.413 | 0.201 | -0.806 | -0.543 | -0.279 | -0.009 |
| B.year[6,1] | 1.001 | -0.195 | 0.199 | -0.587 | -0.328 | -0.062 | 0.199 |
| B.year[7,1] | 1.002 | -0.023 | 0.209 | -0.445 | -0.159 | 0.112 | 0.399 |
| B.year[8,1] | 1.002 | -0.304 | 0.229 | -0.760 | -0.452 | -0.152 | 0.136 |
| B.year[9,1] | 1.000 | -0.145 | 0.227 | -0.588 | -0.297 | 0.003 | 0.291 |
| B.year[10,1] | 1.000 | -0.209 | 0.213 | -0.637 | -0.351 | -0.073 | 0.204 |
| B.year[11,1] | 1.000 | -0.564 | 0.217 | -1.001 | -0.706 | -0.418 | -0.151 |
| B.year[12,1] | 1.000 | 1.036 | 0.199 | 0.656 | 0.907 | 1.166 | 1.436 |
| B.year[13,1] | 1.000 | 0.492 | 0.209 | 0.078 | 0.355 | 0.623 | 0.914 |
| B.year[14,1] | 1.000 | 0.592 | 0.204 | 0.193 | 0.451 | 0.731 | 0.991 |
| B.year[15,1] | 1.000 | 0.621 | 0.214 | 0.222 | 0.476 | 0.764 | 1.042 |
| B.year[1,2] | 1.000 | 1.308 | 0.313 | 0.695 | 1.102 | 1.520 | 1.921 |
| B.year[2,2] | 1.000 | 1.084 | 0.295 | 0.503 | 0.891 | 1.279 | 1.659 |
| B.year[3,2] | 1.000 | 1.145 | 0.294 | 0.552 | 0.952 | 1.344 | 1.713 |
| B.year[4,2] | 1.000 | 2.150 | 0.294 | 1.586 | 1.951 | 2.345 | 2.724 |
| B.year[5,2] | 1.000 | 2.181 | 0.213 | 1.762 | 2.033 | 2.324 | 2.603 |
| B.year[6,2] | 1.000 | 1.223 | 0.180 | 0.869 | 1.102 | 1.347 | 1.567 |
| B.year[7,2] | 1.000 | 1.784 | 0.210 | 1.370 | 1.645 | 1.922 | 2.188 |
| B.year[8,2] | 1.000 | 1.341 | 0.249 | 0.852 | 1.176 | 1.514 | 1.829 |
| B.year[9,2] | 1.000 | 1.629 | 0.246 | 1.143 | 1.471 | 1.792 | 2.101 |
| B.year[10,2] | 1.000 | 1.395 | 0.219 | 0.967 | 1.245 | 1.538 | 1.834 |
| B.year[11,2] | 1.001 | 0.850 | 0.198 | 0.465 | 0.715 | 0.986 | 1.236 |
| B.year[12,2] | 1.000 | 1.682 | 0.161 | 1.364 | 1.570 | 1.794 | 1.986 |
| B.year[13,2] | 1.000 | 1.491 | 0.181 | 1.142 | 1.371 | 1.614 | 1.842 |
| B.year[14,2] | 1.000 | 1.307 | 0.192 | 0.929 | 1.178 | 1.441 | 1.682 |
| B.year[15,2] | 1.000 | 1.471 | 0.217 | 1.038 | 1.327 | 1.614 | 1.895 |
| B.year[1,3] | 1.000 | -2.504 | 0.149 | -2.795 | -2.604 | -2.403 | -2.218 |
| B.year[2,3] | 1.000 | -2.056 | 0.129 | -2.307 | -2.145 | -1.971 | -1.794 |
| B.year[3,3] | 1.000 | -2.527 | 0.191 | -2.909 | -2.651 | -2.400 | -2.151 |
| B.year[4,3] | 1.000 | -2.296 | 0.137 | -2.569 | -2.386 | -2.205 | -2.026 |
| B.year[5,3] | 1.000 | -2.517 | 0.109 | -2.728 | -2.591 | -2.445 | -2.295 |
| B.year[6,3] | 1.001 | -2.370 | 0.087 | -2.543 | -2.428 | -2.312 | -2.195 |
| B.year[7,3] | 1.000 | -2.252 | 0.105 | -2.455 | -2.325 | -2.181 | -2.051 |
| B.year[8,3] | 1.002 | -2.226 | 0.132 | -2.481 | -2.313 | -2.137 | -1.964 |
| B.year[9,3] | 1.000 | -2.272 | 0.134 | -2.536 | -2.362 | -2.179 | -2.013 |
| B.year[10,3] | 1.000 | -2.522 | 0.130 | -2.767 | -2.614 | -2.434 | -2.268 |
| B.year[11,3] | 1.000 | -1.981 | 0.093 | -2.167 | -2.044 | -1.918 | -1.798 |
| B.year[12,3] | 1.000 | -2.422 | 0.073 | -2.567 | -2.471 | -2.369 | -2.279 |
| B.year[13,3] | 1.000 | -2.387 | 0.092 | -2.565 | -2.449 | -2.325 | -2.208 |
| B.year[14,3] | 1.000 | -1.918 | 0.091 | -2.098 | -1.981 | -1.859 | -1.743 |
| B.year[15,3] | 1.000 | -2.447 | 0.107 | -2.657 | -2.520 | -2.373 | -2.241 |
| B.year[1,4] | 1.000 | -0.202 | 0.138 | -0.471 | -0.292 | -0.108 | 0.067 |
| B.year[2,4] | 1.000 | -0.153 | 0.109 | -0.369 | -0.227 | -0.079 | 0.062 |
| B.year[3,4] | 1.000 | 0.195 | 0.138 | -0.063 | 0.101 | 0.284 | 0.473 |
| B.year[4,4] | 1.000 | -0.486 | 0.130 | -0.739 | -0.573 | -0.398 | -0.239 |
| B.year[5,4] | 1.000 | -0.430 | 0.135 | -0.694 | -0.522 | -0.337 | -0.172 |
| B.year[6,4] | 1.000 | 0.069 | 0.089 | -0.101 | 0.008 | 0.131 | 0.240 |
| B.year[7,4] | 1.000 | -0.096 | 0.104 | -0.293 | -0.165 | -0.027 | 0.111 |
| B.year[8,4] | 1.000 | -0.251 | 0.134 | -0.505 | -0.343 | -0.159 | 0.017 |
| B.year[9,4] | 1.000 | 0.199 | 0.146 | -0.086 | 0.102 | 0.294 | 0.492 |
| B.year[10,4] | 1.000 | 0.040 | 0.132 | -0.217 | -0.048 | 0.132 | 0.298 |
| B.year[11,4] | 1.001 | 0.103 | 0.077 | -0.049 | 0.050 | 0.155 | 0.253 |
| B.year[12,4] | 1.000 | -0.382 | 0.057 | -0.491 | -0.421 | -0.343 | -0.272 |
| B.year[13,4] | 1.000 | -0.086 | 0.079 | -0.238 | -0.141 | -0.031 | 0.064 |
| B.year[14,4] | 1.000 | -0.248 | 0.083 | -0.410 | -0.303 | -0.191 | -0.086 |
| B.year[15,4] | 1.000 | -0.101 | 0.105 | -0.302 | -0.171 | -0.031 | 0.111 |
| ar1[1] | 1.000 | 0.789 | 0.012 | 0.765 | 0.781 | 0.797 | 0.814 |
| ar1[2] | 1.000 | 0.771 | 0.018 | 0.734 | 0.759 | 0.784 | 0.800 |
| ar1[3] | 1.001 | 0.801 | 0.013 | 0.777 | 0.791 | 0.810 | 0.827 |
| ar1[4] | 1.000 | 0.767 | 0.019 | 0.726 | 0.755 | 0.781 | 0.799 |
| ar1Mean | 1.001 | 0.788 | 0.038 | 0.734 | 0.773 | 0.795 | 0.907 |
| ar1SD | 1.005 | 0.047 | 0.063 | 0.002 | 0.014 | 0.050 | 0.245 |
| mu.year[2], Linear | 1.001 | 1.471 | 0.153 | 1.173 | 1.370 | 1.569 | 1.786 |
| mu.year[3], Quadratic | 1.001 | -2.314 | 0.103 | -2.517 | -2.382 | -2.246 | -2.118 |
| mu.year[4], Cubic | 1.000 | -0.123 | 0.100 | -0.318 | -0.189 | -0.060 | 0.076 |
| sigma | 1.000 | 0.597 | 0.004 | 0.588 | 0.594 | 0.600 | 0.606 |
| sigma.b.year[1] | 1.000 | 0.580 | 0.123 | 0.394 | 0.494 | 0.646 | 0.861 |
| sigma.b.year[2] | 1.000 | 0.501 | 0.113 | 0.327 | 0.420 | 0.563 | 0.764 |
| sigma.b.year[3] | 1.000 | 0.349 | 0.070 | 0.239 | 0.300 | 0.388 | 0.516 |
| sigma.b.year[4] | 1.000 | 0.357 | 0.071 | 0.245 | 0.307 | 0.397 | 0.527 |
