# Supplementary figures and images for: A hierarchical model of daily stream temperature using air-water temperature synchronization, autocorrelation, and time lags
Source: PeerJ. 2016 Feb 29;4:e1727. doi: 10.7717/peerj.1727 (PMC4782734; doi:10.7717/peerj.1727)

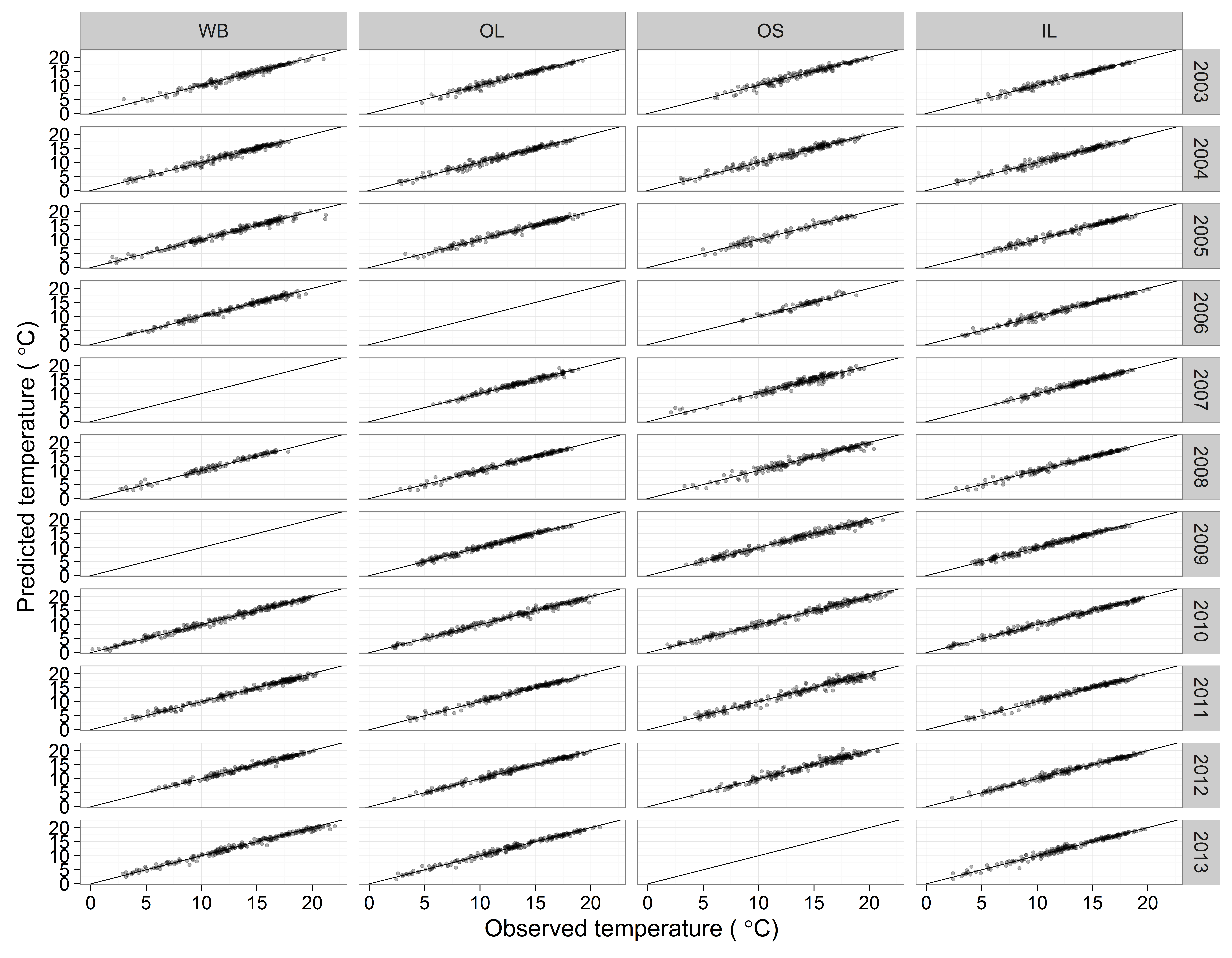

Supplement: Figure S2 — Observed vs. predicted daily water temperatures for combinations of site and year. The line in each panel is the 1:1 line. [file peerj-04-1727-s005.png]

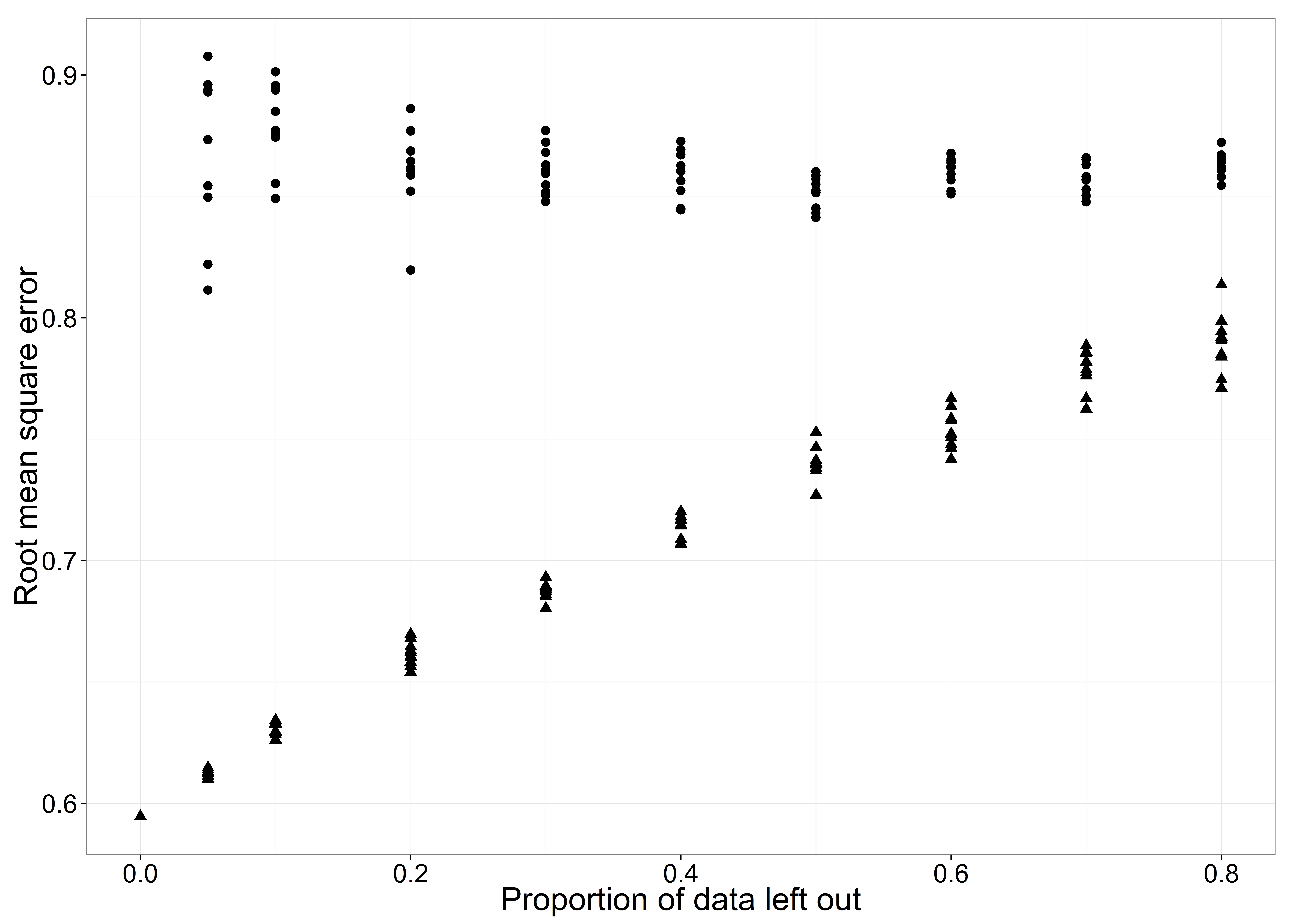

Supplement: Figure S3 — Root mean square error of the test data set (triangles) and training data set (circles) for 10 replicates of runs with increasing proportions of data left out of the training set. [file peerj-04-1727-s006.png]

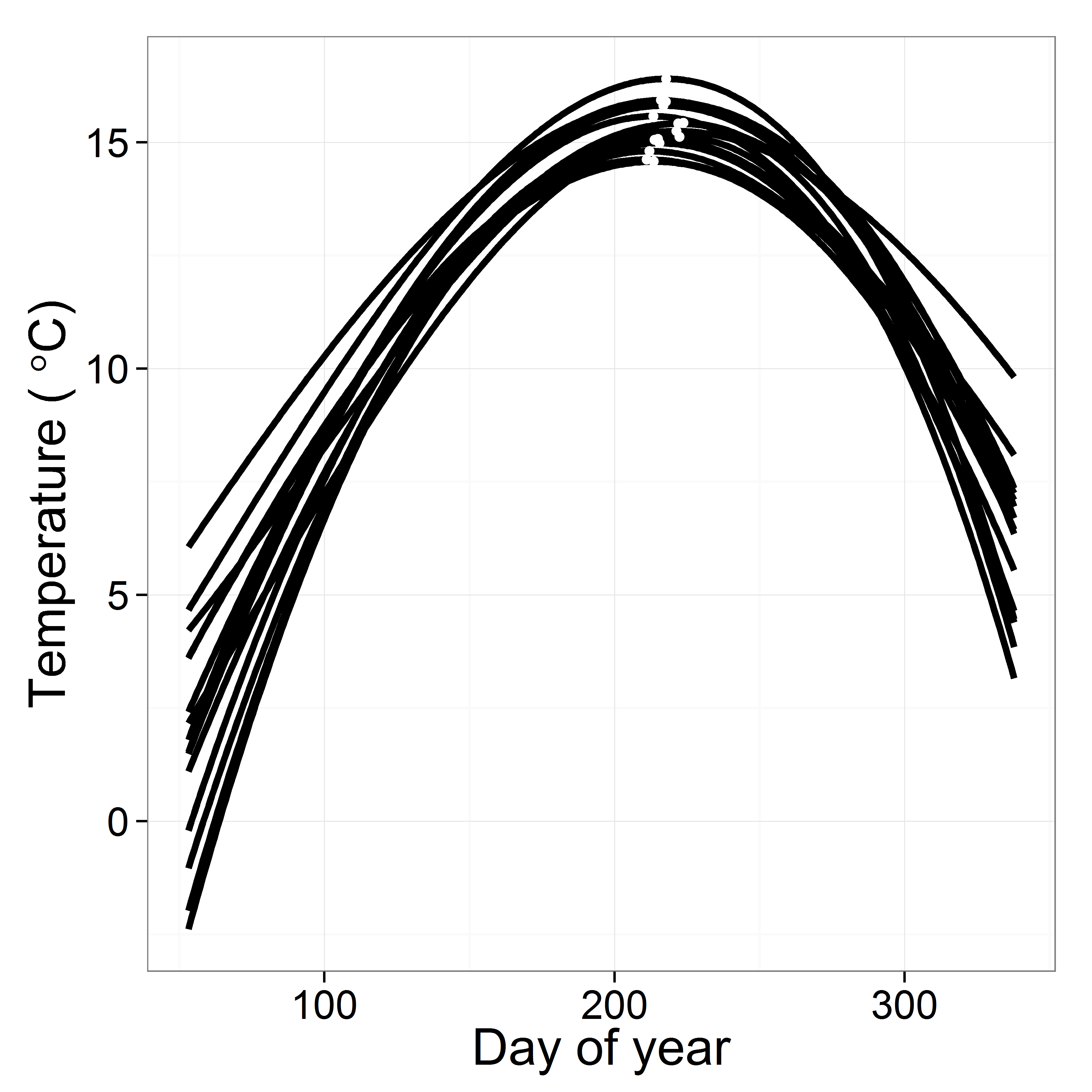

Supplement: Figure S4 — Predicted cubic curves for each year against day of year (Eq. (7)). The white dots signify the yearly maximum water temperature and the day of maximum water temperature. [file peerj-04-1727-s007.png]
